# Pleuroparenchymal fibroelastosis associated with anti-Ku antibody-positive polymyositis: A case report with prominent systemic muscle atrophy and review of five cases

**DOI:** 10.1016/j.rmcr.2026.102383

**Published:** 2026-02-06

**Authors:** Yusuke Sakaue, Ryosuke Hanaoka

**Affiliations:** a Department of Rheumatology and Internal Medicine, Kamitsuga General Hospital, 1-1033, Shimodamachi, Kanuma, Tochigi, 322-8550, Japan; b Department of Rheumatology, NHO Tochigi Medical Center, 1-10-37, Nakatomatsuri, Utsunomiya, Tochigi, 320-8580, Japan

**Keywords:** Pleuroparenchymal fibroelastosis, Anti-Ku antibody, Polymyositis, Muscle atrophy, Cyclophosphamide, Restrictive lung disease

## 1 Introduction

Pleuroparenchymal fibroelastosis (PPFE) is a rare form of interstitial lung disease characterized by upper-lobe–predominant subpleural fibrosis, pleural thickening, and marked flattening of the thoracic cage. Since its initial clinicopathologic description by Frankel et al. [1], PPFE has been increasingly recognized as a distinct disease entity within the spectrum of idiopathic interstitial pneumonias. Although the etiology remains incompletely understood, several clinical contexts have been associated with PPFE, including prior hematopoietic stem cell transplantation, exposure to alkylating agents such as cyclophosphamide, chronic hypersensitivity pneumonitis, nontuberculous mycobacterial infection, and connective tissue diseases [[2], [3], [4], [5]].

Autoimmune mechanisms have been suspected in a subset of PPFE cases, as nonspecific autoantibodies are frequently detected and connective tissue diseases are disproportionately represented among affected individuals [6,7]. However, the specific autoantibody profiles associated with PPFE have not been well defined.

Anti-Ku antibody is present in approximately 5–10% of patients with polymyositis/dermatomyositis and is typically associated with an overlap syndrome characterized by myositis, interstitial lung disease (ILD), Raynaud's phenomenon, arthritis, and scleroderma-like features [8,9]. The ILD pattern most commonly linked to anti-Ku antibody is nonspecific interstitial pneumonia (NSIP) or, less frequently, usual interstitial pneumonia (UIP), both of which usually show lower-lobe predominance [10]. To date, an association between anti-Ku antibody–positive myositis and PPFE—an upper-lobe–dominant fibrotic process—has not been reported.

Here, we describe a patient with anti-Ku antibody–positive polymyositis who developed radiologically and physiologically typical PPFE, along with profound ventilatory impairment. In addition, we summarize four further cases of PPFE encountered in our institution. This report highlights an unrecognized association between anti-Ku–positive myositis and PPFE and expands the clinical spectrum of autoimmune diseases complicated by PPFE.

## 2 Methods

This study was conducted using a retrospective review of medical records at the National Hospital Organization Tochigi Medical Center. The index case and four additional patients with pleuroparenchymal fibroelastosis (PPFE) were identified through a keyword-based search of institutional radiology reports (using terms including “PPFE,” “pleuroparenchymal fibroelastosis,” “upper-lobe fibrosis,” and “platythorax”), supplemented by clinician recall of cases meeting the diagnostic criteria. No systematic HRCT database screening was performed; therefore, ascertainment bias cannot be excluded, and some cases may not have been captured. The review period spanned from April 2020 to March 2024. Clinical information—including pulmonary function tests, arterial blood gases, laboratory data, radiologic studies, and treatment history—was extracted from electronic medical records.

The diagnosis of PPFE was established according to the 2019 Japanese diagnostic criteria [3], requiring (1) upper-lobe–predominant subpleural fibrosis with pleural thickening on high-resolution computed tomography (HRCT), (2) evidence of disease progression, and (3) exclusion of other identifiable causes. When available, pathological findings were also reviewed; one case underwent autopsy for histologic confirmation.

## 3 Case presentation

A 45-year-old woman presented with progressive exertional dyspnea. She had noticed slowly progressive proximal muscle weakness since 2005, and by 2010 she became unable to stand from a sitting position. Laboratory examination revealed elevated muscle enzymes (CK 2055 IU/L, myoglobin 350 ng/mL, aldolase 19.3 IU/L) and a positive antinuclear antibody (1:640, speckled and nucleolar pattern). Anti-Ku antibody was strongly positive (3+ by immunoblot), and anti-SSA antibody was elevated at 30.0 U/mL. MRI of the thighs showed diffuse high signal intensity in the rectus femoris and vastus lateralis muscles, and needle electromyography demonstrated myotonic discharges resembling a “dive-bomber” pattern. Muscle biopsy revealed severe fatty replacement without inflammatory infiltrates. Chest CT showed mild bilateral interstitial infiltrates.

Based on these findings, she was diagnosed with polymyositis and connective tissue disease-associated interstitial lung disease (CTD-ILD). Initial therapy with prednisolone (60 mg/day) and azathioprine (100 mg/day) was started, followed by multiple immunosuppressive agents including cyclophosphamide (four cycles of intravenous pulse therapy), cyclosporine, tacrolimus, mizoribine, mycophenolate mofetil, tofacitinib, and intravenous immunoglobulin therapy, none of which achieved satisfactory control.

Abatacept (500 mg monthly) was introduced in January 2019. However, her dyspnea worsened around January 2020, accompanied by a rise in KL-6 levels. Following relocation to her hometown during the COVID-19 pandemic, her symptoms progressed with further muscle weakness, weight loss, anorexia, and mediastinal emphysema. She was hospitalized in April 2022 for further evaluation. During hospitalization, she experienced recurrent bilateral pneumothorax. Atrial fibrillation developed, for which edoxaban was initiated. Because of prolonged mechanical ventilation dependency, she was transferred to our hospital in September 2023.

On admission to our hospital, her height was 157 cm and weight 38.8 kg (BMI 15.7). Marked emaciation with flattening of the thorax and intercostal muscle atrophy was noted. Breath sounds were diminished with fine crackles in the bilateral lower lung fields. Laboratory findings revealed hypoproteinemia (TP 6.9 g/dL), hypoalbuminemia (Alb 3.8 g/dL), and elevated surfactant protein-D (SP-D, 126 ng/mL), while KL-6 remained within the upper normal limit (551 IU/L). Immunologic testing was positive for ANA (1:1280), anti-SSA, and anti-Ku antibodies. Arterial blood gas analysis on room air showed severe hypercapnia (PaCO_2_ 62.6 Torr). Pulmonary function testing demonstrated severely reduced lung volumes (VC 320 mL, 10.5% predicted; TLC 800 mL, 19.6% predicted) and a markedly elevated residual volume ratio (RV/TLC 60%, 217.9% predicted). Cerebrospinal fluid analysis showed elevated lactate (23.3 mg/dL, normal <22) and pyruvate (1.07 mg/dL, normal <0.16), suggesting mitochondrial metabolic disturbance. Electromyography demonstrated myogenic changes without fasciculation potentials. Muscle biopsy revealed mild fiber size variation and diffuse HLA-ABC expression, compatible with immune-mediated necrotizing myopathy (Fig. 3). Ragged-red fibers were absent.

**Fig. 1:**
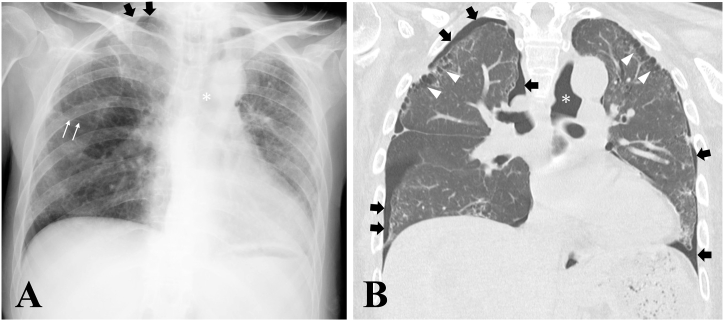
**Chest radiograph and computed tomography findings** (A) Chest radiograph showing bilateral upper lobe volume loss with hilar elevation and platythorax. (B) Coronal chest CT demonstrating bilateral upper-lobe-dominant subpleural fibrosis with honeycombing and traction bronchiectasis. Thin white arrows indicate elevated fissures due to upper lobe volume loss; thick black arrows indicate pneumothorax; asterisks indicate pneumomediastinum; white arrowheads indicate bilateral upper-lobe-dominant subpleural fibrosis with honeycombing.

**Fig. 2:**
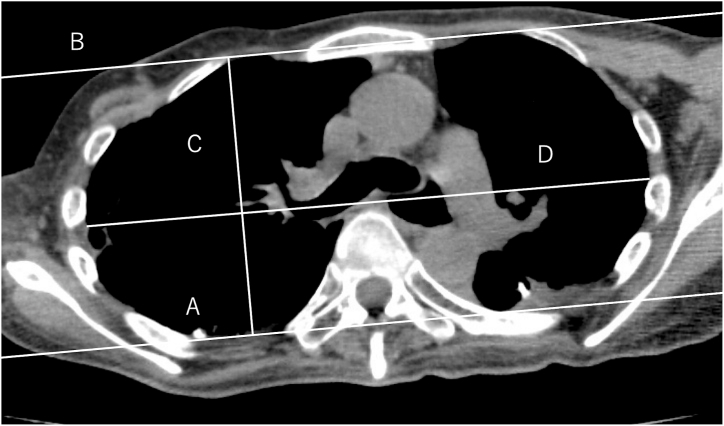
**Measurement of thoracic flattening** Axial chest CT at the sixth thoracic vertebra level showing marked flattening of the thoracic cage. The flattening index (anteroposterior diameter/transverse diameter) was 0.501, indicating severe platythorax. Lines A and B represent the posterior and anterior borders of the bony thorax, respectively. C indicates the anteroposterior diameter, and D indicates the maximum transverse diameter.

Chest imaging studies demonstrated characteristic findings of PPFE. The chest radiograph showed bilateral upper lobe volume loss with hilar elevation and platythorax (Fig. 1A). Chest CT demonstrated bilateral upper-lobe-dominant subpleural fibrosis with honeycombing, traction bronchiectasis, and ground-glass opacities (Fig. 1B). The flattening index, calculated at the sixth thoracic vertebral level as the ratio of anteroposterior diameter to transverse diameter, was 0.501 (normal >0.65 in healthy individuals; values ≤ 0.63 suggest PPFE [3]), confirming marked flattening of the thoracic cage (Fig. 2). The patient fulfilled the diagnostic criteria for PPFE, including progressive dyspnea, upper-lobe-predominant subpleural fibrosis on CT, thoracic flattening, elevated RV/TLC ratio, and severe cachexia.

**Fig. 3:**
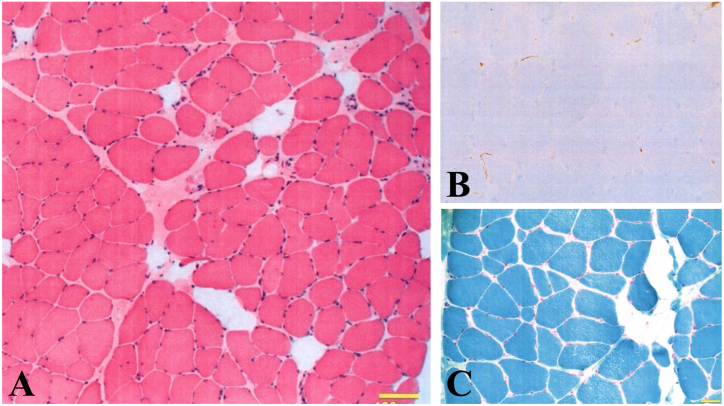
**Muscle biopsy findings** (A) Hematoxylin-eosin staining showing moderate fiber size variation. (B) HLA-ABC immunostaining demonstrating diffuse sarcolemmal expression. (C) Gomori trichrome staining showing absence of ragged-red fibers. These findings are compatible with immune-mediated necrotizing myopathy. Scale bar = 100 μm.

Despite immunosuppressive therapy, ventilatory impairment continued to progress. After stabilization, the patient was discharged and remained at home for 10 months; however, chronic respiratory failure gradually worsened, ultimately necessitating transfer to a long-term care facility because of full dependence for activities of daily living.

We have encountered four additional cases of PPFE at our institution, all demonstrating similar systemic manifestations including severe cachexia (mean BMI 15.95), marked air trapping (mean RV/TLC 164.7% predicted), severe hypercapnia (mean PaCO_2_ 66.04 Torr), and progressive limb weakness (Table 1). Three of five cases were associated with underlying autoimmune diseases.

**Table 1:** Clinical characteristics of five PPFE cases at our institution All five patients demonstrated consistent systemic manifestations including severe cachexia (mean BMI 15.95 kg/m^2^), marked air trapping (mean RV/TLC 164.7% predicted), severe hypercapnia (mean PaCO2 66.04 Torr), progressive limb weakness, anorexia, and recurrent pneumothorax. Three of five cases (60%) were associated with underlying autoimmune diseases. These findings suggest that PPFE may represent a systemic disorder rather than a disease confined to the lungs. Abbreviations: BMI, body mass index; F, female; M, male; NA, not available; PaCO2, partial pressure of arterial carbon dioxide; PPFE, pleuroparenchymal fibroelastosis; RV, residual volume; TLC, total lung capacity.

Case	Age (years)	Sex	BMI (kg/m^2^)	RV/TLC(% predicted)	PaCO2 (Torr)	Dyspnea	Anorexia	Limb Weakness	Pneumothorax	Underlying Autoimmune Disease
Case 1	82	F	15.78	150	45.6	++	+	+	+	None
Case 2	74	F	19.48	NA	68	++	+	+	++ (recurrent)	None
Case 3	84	F	15.52	NA	69.8	++	+	+	+	Sjögren's syndrome
Case 4	73	M	13.27	126.2	84.2	++	+	+	+	Antisynthetase syndrome
Our case	45	F	15.7	217.9	62.6	++	+	+	++ (recurrent)	Anti-Ku antibody-positive polymyositis
Summary	Mean: 71.6	4F/1M	Mean: 15.95	Mean: 164.7∗	Mean: 66.04	100% (5/5)	100% (5/5)	100% (5/5)	100% (5/5)	60% (3/5)

Abbreviations: F, female; M, male; RV/TLC, Rsidual Volume/Total Lung Capacity; +, present; ++, severe ∗Mean calculated from three cases with available data (Cases 1, 4, and index case).

Given the coexistence of anti-Ku antibody-positive polymyositis, characteristic radiologic and physiological findings of PPFE, and progressive systemic muscle atrophy possibly involving mitochondrial dysfunction, we diagnosed this patient with pleuroparenchymal fibroelastosis secondary to polymyositis with systemic muscle involvement.

## 4 Discussion

We describe a case of anti-Ku antibody–positive polymyositis complicated by pleuroparenchymal fibroelastosis (PPFE), accompanied by profound systemic muscle atrophy including respiratory muscles. Although the patient exhibited classical radiologic hallmarks of PPFE—upper-lobe–predominant subpleural fibrosis, pleural thickening, platythorax, and markedly elevated RV/TLC ratio—her clinical course was also characterized by severe cachexia, progressive limb weakness, and chronic hypercapnic respiratory failure. These extrapulmonary manifestations were similarly observed across four additional PPFE cases identified at our institution, suggesting that systemic involvement may occur in a subset of patients with PPFE. However, given the retrospective nature of this series and its small sample size, these observations should be interpreted as hypothesis-generating rather than indicating causality.

PPFE is a rare subtype of interstitial pneumonia exhibiting progressive upper-lobe fibrosis and pleural elastosis [[1], [2], [3], [4], [5]]. It has been associated with a wide range of clinical contexts, including hematopoietic stem cell transplantation, chronic hypersensitivity pneumonitis, connective tissue diseases, nontuberculous mycobacterial infection, Aspergillus infection, and exposure to alkylating agents such as cyclophosphamide. Although the pathogenesis remains unclear, autoimmune mechanisms and treatment-related lung injury have been proposed [[4], [5], [6], [7]]. Our patient received four cycles of cyclophosphamide, and while this may have contributed to PPFE development, a direct causal link cannot be established.

Anti-Ku antibodies are detected in approximately 5–10% of patients with inflammatory myopathies and are typically associated with overlap syndromes characterized by myositis, interstitial lung disease—usually with NSIP or UIP patterns—and features of scleroderma or lupus [[8], [9], [10]]. The presence of anti-Ku antibodies in a patient who developed PPFE, a disease with distinct upper-lobe predominance, is therefore notable. To our knowledge, no prior reports have specifically described PPFE occurring in the setting of anti-Ku–positive polymyositis. Enomoto et al. reported two cases of PPFE associated with systemic sclerosis and polymyositis, but neither was positive for anti-Ku antibody [6].

A distinctive feature of the present case and our institutional series is the prominence of extrapulmonary manifestations. While weight loss and low BMI are mentioned in prior PPFE descriptions, the severity of cachexia, hypercapnia, and generalized muscle weakness in our patients appeared disproportionate to the degree of pulmonary restriction alone. Several alternative explanations should be carefully considered. Advanced respiratory failure itself can lead to chronic hypercapnia, disuse atrophy, and secondary malnutrition. Long-term corticosteroid therapy and chronic inflammation may contribute to muscle wasting [11,12]. Chronic inflammation, reduced caloric intake, and increased metabolic demand may also exacerbate muscle loss. Therefore, although our observations raise the possibility that PPFE may have systemic components, direct causation cannot be concluded from the available evidence.

In our index patient, elevated cerebrospinal fluid lactate and pyruvate suggested a possible disturbance in oxidative metabolism. This prompted consideration of mitochondrial dysfunction as a contributing factor to her profound muscle atrophy and ventilatory insufficiency. However, the absence of ragged-red fibers on muscle biopsy and the unavailability of mitochondrial DNA analysis preclude any definitive conclusion. CSF lactate evaluation is not routinely performed in inflammatory myopathies; in this case, it was obtained to investigate unexplained hypercapnia and progressive weakness out of proportion to creatine kinase activity. Future studies are needed to clarify whether mitochondrial impairment plays a role in selected PPFE cases. It is conceivable that chronic hypoxia and hypercapnia associated with severe restrictive lung disease may lead to secondary mitochondrial dysfunction, creating a vicious cycle of progressive muscle weakness and respiratory failure.

The four additional PPFE cases identified at our institution were collected through a retrospective chart review conducted between 2020 and 2024, and each fulfilled the 2019 Japanese diagnostic criteria for PPFE [3]. While these cases displayed similar systemic manifestations, the retrospective design, small sample size, and heterogeneity of underlying autoimmune diseases limit generalizability. Furthermore, only one patient underwent pathological confirmation through autopsy; PPFE diagnosis in the remaining cases relied primarily on high-resolution CT findings, as surgical lung biopsy is often not feasible in patients with poor respiratory reserve [3]. These methodological constraints should temper the interpretation of systemic involvement patterns.

The prognosis of PPFE is generally poor, with progressive respiratory failure being the most common cause of death. There is no established effective treatment, although immunosuppressive therapy, antifibrotic agents such as nintedanib and pirfenidone, and lung transplantation have been attempted with variable success. In our patient, despite multiple immunosuppressive therapies, disease progression was relentless. The coexistence of severe cachexia and muscle atrophy further complicates management, as nutritional support and rehabilitation become critically important but challenging in the setting of severe respiratory compromise.

Given the poor prognosis of PPFE and the lack of established treatments, comprehensive multidisciplinary management is essential. This includes not only consideration of immunosuppressive and antifibrotic therapies but also attention to nutritional support, pulmonary rehabilitation, and management of complications such as pneumothorax and respiratory failure. Early referral for lung transplantation evaluation may be appropriate for selected patients.

Taken together, this case highlights the potential coexistence of PPFE and anti-Ku–positive polymyositis and underscores the importance of evaluating both pulmonary and extrapulmonary manifestations in patients with suspected PPFE. Although our findings suggest that systemic muscle atrophy, metabolic abnormalities, and respiratory muscle dysfunction may contribute to the severity of respiratory impairment in some PPFE patients, confirmation will require larger, prospective studies with detailed neuromuscular assessment.

Our study has several limitations. First, we were unable to measure latent TGF-β binding protein-4 (LTBP-4), a recently identified biomarker that correlates with disease activity in PPFE. Second, the diagnosis of mitochondrial myopathy could not be confirmed due to the lack of mitochondrial DNA analysis and electron microscopy.

## CRediT authorship contribution statement

**Yusuke Sakaue:** Writing – original draft. **Ryosuke Hanaoka:** Writing – review & editing, Validation.

## Ethics approval and consent to participate

Ethical approval was not required for this case report in accordance with institutional and national guidelines.

## Consent for publication

Written informed consent was obtained from the patient for publication of this case report and accompanying images. A copy of the written consent is available for review by the Editor-in-Chief of this journal.

## Availability of data and materials

All data relevant to this case report are included in the article. Additional clinical records are available from the corresponding author upon reasonable request and with appropriate patient consent.

## Funding

The authors received no specific grant from any funding agency in the public, commercial, or not-for-profit sectors for this case report.

## Declaration of competing interest

The authors declare that they have no known competing financial interests or personal relationships that could have appeared to influence the work reported in this paper.
